# Intelligence in Williams Syndrome Is Related to *STX1A*, Which Encodes a Component of the Presynaptic SNARE Complex

**DOI:** 10.1371/journal.pone.0010292

**Published:** 2010-04-21

**Authors:** Michael C. Gao, Ursula Bellugi, Li Dai, Debra L. Mills, Eric M. Sobel, Kenneth Lange, Julie R. Korenberg

**Affiliations:** 1 Medical Genetics Institute, Cedars-Sinai Medical Center, Los Angeles, California, United States of America; 2 Department of Human Genetics, University of California Los Angeles, Los Angeles, California, United States of America; 3 Department of Pediatrics, University of California Los Angeles, Los Angeles, California, United States of America; 4 Salk Institute Laboratory for Cognitive Neuroscience, La Jolla, California, United States of America; 5 Center for Integrated Neurosciences and Human Behavior, The Brain Institute, Department of Pediatrics, University of Utah, Salt Lake City, Utah, United States of America; 6 School of Psychology, Bangor University, Bangor, Gwynedd, United Kingdom; 7 Departments of Biomathematics and Statistics, University of California Los Angeles, Los Angeles, California, United States of America; Ohio State University Medical Center, United States of America

## Abstract

Although genetics is the most significant known determinant of human intelligence, specific gene contributions remain largely unknown. To accelerate understanding in this area, we have taken a new approach by studying the relationship between quantitative gene expression and intelligence in a cohort of 65 patients with Williams Syndrome (WS), a neurodevelopmental disorder caused by a 1.5 Mb deletion on chromosome 7q11.23. We find that variation in the transcript levels of the brain gene *STX1A* correlates significantly with intelligence in WS patients measured by principal component analysis (PCA) of standardized WAIS-R subtests, r  = 0.40 (Pearson correlation, Bonferroni corrected p-value  = 0.007), accounting for 15.6% of the cognitive variation. These results suggest that syntaxin 1A, a neuronal regulator of presynaptic vesicle release, may play a role in WS and be a component of the cellular pathway determining human intelligence.

## Introduction

Intelligence is a largely heritable, quantitative trait that varies from mild mental retardation to highly gifted [1]. Despite decades of intensive research, there are few proven links between genes and cognitive function, none explaining more than a few percent of cognitive variation [2], [3], [4]. Human intelligence is measured by a series of tests that have been standardized in the normal population using the Intelligence Quotient (IQ), as defined by the Wechsler Adult Intelligence Scale-Revised (WAIS-R), which is determined by 11 subtests grouped into two categories, one verbal IQ (VIQ) and the other visual-spatial or performance IQ (PIQ) [5].

**Figure 1:**
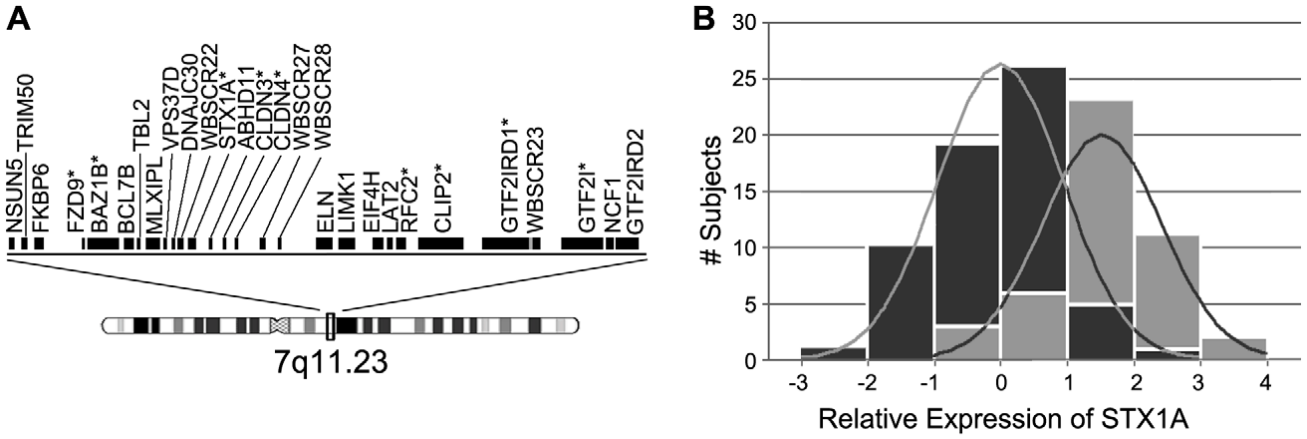
Distribution of quantitative transcription of genes deleted in WS. **a: Map of genes commonly deleted in WS.** Genes analyzed in this report are indicated with an asterisk. **b: Z-scores (relative to the WS mean) of** ***STX1A*** **expression levels.** *STX1A* measured by quantitative RT-PCR of lymphoblast cDNA in 62 WS subjects [14] (dark gray) and 45 normal controls [14] (light gray). The expression level distributions of the remaining WS-deleted genes are shown in Figure S1.

Neurodevelopmental disorders such as Williams Syndrome (WS) offer a unique opportunity to probe the connections between genes and IQ in that WS is caused by deletion of about 28 genes located in a 1.5 Mb region on chromosome 7q11.23. Williams Syndrome presents with a distinct pattern of intellectual disabilities that differ from normal on subtests of the WAIS-R. In general, WS cases exhibit relative peaks in verbal ability and valleys in visual-spatial processing [6], [7], [8]. Specifically, relative to their overall performance, WS subjects tend to do well in tests of vocabulary (Vocabulary) and abstract reasoning (Similarities, Picture Arrangement), and poorly in tests of numeracy (Arithmetic), visual-spatial (Digit Symbol, Block Design, Object Assembly), and memory (Digit Span) [6]. Therefore, the IQ determined by the WAIS-R, standardized in the normal population, may not optimally reflect variations in cognitive function in those with WS, limiting the ability to discern correlations with genetic variation. The breakpoints that are clustered in the regions of highly repetitive DNA segments that flank the WS deletion further constrain the power to resolve genetic contributions to WS cognition. These breakpoints result in hemizygosity for the same set of genes in the majority of WS cases (Fig. 1). Therefore, a major question in the field has been the genetic causes of the cognitive variation found in typical WS.

To begin to correlate cognitive deficits with specific deleted genes, we and others have studied rare WS individuals with smaller deletions [7], [8], [9], [10], [11]. This leads to the suggestion of a role for *GTF2I* and *GTF2IRD1* in the visual-spatial construction and associated neuroanatomical defects of posterior cortices seen in WS patients [7], [11]. However, the number of atypical deletions is small and has limited such analyses. Another potential source of genetic variation in WS is the level of activity of the genes remaining on the non-deleted chromosome 7. That is, with a deletion of chromosome 7q11.23, persons with WS lose one copy and remain with only a single copy of the genes in this region. It is the decreased or altered ability of this single remaining gene copy to generate normal transcription that is ultimately responsible for the features of WS. Although variation in locus specific gene expression has been related to disease risk [12], this has been neglected as a source of variation in WS. The current report addresses the problem of genetic variation in WS by using quantitative gene expression and principal components analysis (PCA) to investigate how genetic variation is related to cognition in the vast majority of WS subjects with typical deletions.

Our basic hypothesis is that the severity of cognitive deficits is related to the expression levels of the WS region genes that remain on the normal chromosome 7 that is inherited. Since measuring gene expression in the tissue of interest (brain) is not possible, we and others [13], [14] have quantitated gene expression in lymphoblastoid (LB) cell lines. Clearly there are tissue specific patterns of gene expression that differentiate brain (or other tissues affecting intelligence) and lymphoid tissues. Thus, expression in lymphoblastoid cells may not be well correlated with gene expression levels in cells that directly impact intelligence. Further, the cellular processes of lymphoblastoid cells may be more or less related to brain tissue functions than are typical, non-transformed lymphocytes. Nonetheless, the use of lymphoblastoid cells is supported by previous work showing lymphoblast and lymphocyte gene expression to be altered in fragile X and dup15q autism subtypes [15], as well as in other neuropsychiatric diseases such as schizophrenia, depression, stress and anxiety, and Alzheimer disease [16], [17], [18]. Interestingly, lymphocytes have voltage-gated calcium channel subunits typically limited to excitable cells [19], [20], and respond to neurotransmitters such as GABA GAT-1 [21], [22], dopamine [23], serotonin [24], vasopressin [25], and epinephrine/norepinephrine [26]. We therefore considered the possibility that a subset of WS genes might act in cellular processes that affect the development and function of both the brain and the immune system.

**Figure 2:**
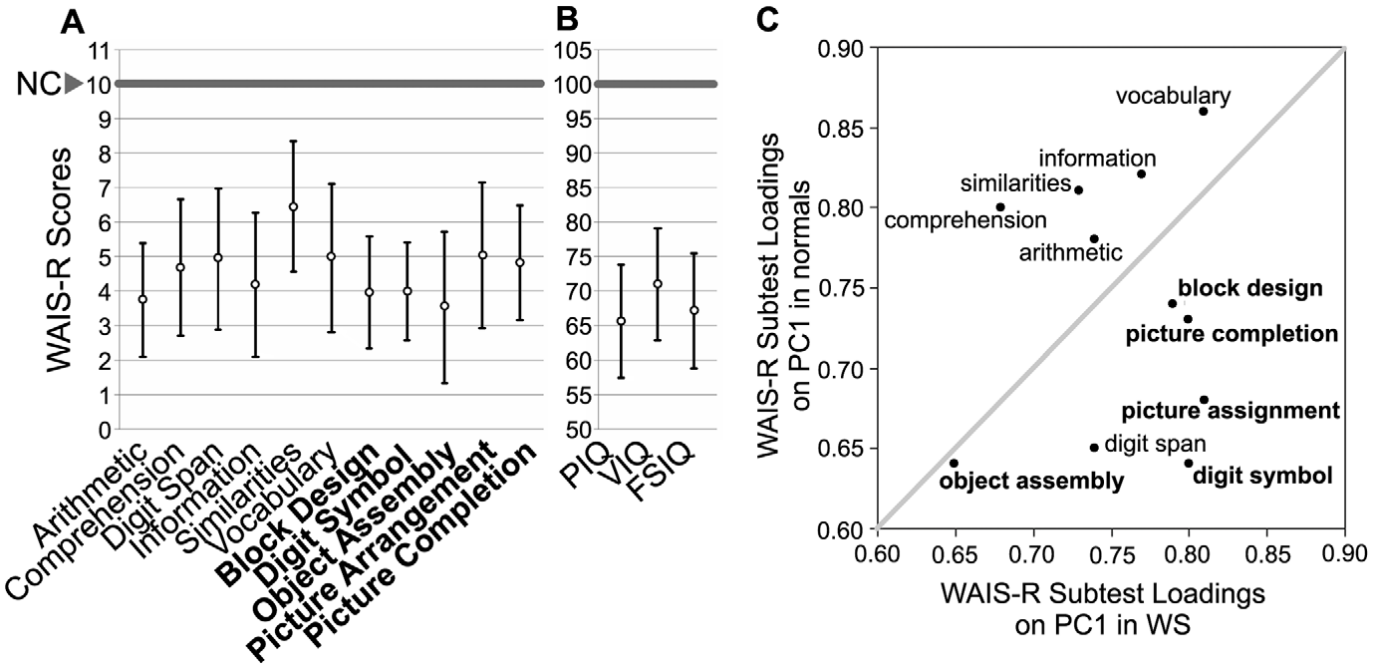
Cognitive performance in WS subjects (n = 67) versus normal controls. **a: Means (circles) and SD (bars) of WAIS-R subtest scores for WS cases.** Performance subtests are listed in bold; verbal subtests in plain font. In normal controls (NC), WAIS-R subtest scores are 10±3 (thick gray line). **b: Means (circles) and SD (bars) of VIQ, PIQ, and FSIQ scores for WS cases.** In normal controls (NC), WAIS-R VIQ, PIQ, and FSIQ scores are 100±15 (thick gray line). **c: Principal component analysis of WAIS-R shows subtests contribute differently to intelligence in WS cases versus normal controls.** WAIS-R subtest loadings on PC1 in WS cases (x-axis) and normal controls (y-axis). Data used for normal analysis is from Enns and Reddon [27]. Performance subtests are listed in bold; verbal subtests in plain font. The iagonal line is x  =  y, at which WS and normal loadings are equal.

WS cognition is distinctive, and one challenge has been to reflect the distinctive peaks and valleys of WS brain and cognitive patterns using IQ subtests standardized on normal individuals (Fig. 2). To address this, we used principal component analysis (PCA) to identify the unique subtest patterns of the WAIS-R found in WS. PCA analysis looks at the correlations of all subtests and seeks to capture most of the observed variation across the tests by a small number of linear combinations of the subtests. The first and most important principal component (PC1) of WAIS-R subtests determined in the normal population is mostly weighted (or loaded) on verbal ability. Because this verbal predominance may be false for WS patients, IQ calculated in the usual way may not optimally reflect the variation in WS cognitive function. Therefore, PCA may more accurately model the distinct cognitive profile of WS than do standard IQ summary measures. Since PCA can generate a single measure of intellectual performance, it sidesteps many of the multiple testing issues that arise in dealing with the full multiplicity of subtests.

In the current study of a large cohort with WS (n = 65), we correlate cognitive performance as reflected in PCA with quantitative expression levels of 10 genes located in the WS deletion, determined by quantitative RT-PCR. We find that the relative expression of the *STX1A* gene accounts for 15.6% of the variance in the first principal component of the WAIS-R in WS cases. After correcting for multiple tests, the evidence clearly indicates that expression levels of *STX1A* partially predict intelligence in WS. Whether *STX1A* exerts a similar influence in normal controls and whether the effect is due to genetic variation of *STX1A* (cis effects) or to regulators in the remainder of the genome (trans effects) is unknown. However, our results suggest that monitoring gene expression may provide unique insights into the neurobiology and genetics of intelligence in WS subjects and possibly the normal population.

## Results

Our approach and hypotheses incorporate three crucial strategies: (i) sensitive, quantitative analysis of gene expression, (ii) principal components analysis of cognitive test scores to better reflect WS cognition, and (iii) correlation of cognitive performance with WS gene expression within a cohort of WS cases with typical deletions.

### Gene Expression

Gene expression was analyzed using quantitative RT-PCR, as described previously [14]. Briefly, subjects' lymphoblastoid cell lines were grown under controlled conditions. To assess WS-region gene transcript levels, we isolated mRNA and performed quantitative RT-PCR on *FZD9*, *BAZ1B*, *STX1A*, *CLDN3*, *CLDN4*, *RFC2*, *CLIP2*, *GTF2IRD1* (exon 2–3), *GTF2IRD1* (exon 10–11), and *GTF2I* for 107 WS subjects (see map of deleted genes in WS in Fig. 1a). We then calibrated the expression levels across subjects using the comparative Ct method with *ACTB*, *PPIA*, and *HPRT1* as reference (control) genes. The reference genes (*ACTB*, *PPIA*, and *HPRT1*) for WS gene expression were chosen in the previous study [14] because two are on chromosome 7 (*ACTB* and *PPIA*), all three were expressed in lymphocytes or lymphoblastoid cell lines, and their expression levels were in the range of the WS genes measured in normal controls. *ACTB* is highly and somewhat variably expressed. *HPRT1* is located on the X chromosome, which could potentially generate additional variability, and was therefore not used for the PC1 correlations. However, the ΔCt's for each WS gene, normalized with each of the three controls, follow a pattern similar to that seen in [14]. No difference in the expression of control genes was found in WS cases versus normal controls, although this is another potential source of variation. The ΔCt's for WS genes normalized with ACTB are given in Supplemental Table 2 of Collette, et al. [14], for both controls and WS cases. For the current paper, we identified a sub-cohort of 65 subjects who had gene expression and WAIS-R data available. Of these 65, probes failed in 3 subjects for *STX1A* normalized to both *ACTB* and *PPIA*, resulting in 62 available subjects. This does not affect our results because the gene expression experiment was done blind to their cognitive scores. For this population, we calculated gene expression Z-scores, i.e., the number of standard deviations by which an observation differs from the population mean. The expression values used in this paper are the average of the *ACTB*-controlled and *PPIA*-controlled Z-scores, as shown for *STX1A* in Fig. 1b and for other genes in Fig. S1. Quantitative PCR was used to confirm that deletions in all 65 WS cases included *STX1A*, but all 10 normal controls included both copies of *STX1A* (Fig. S2).

### Measurement of WS Cognition I: WAIS-R

Williams Syndrome patients have mild intellectual deficiencies but show a distinct pattern of weaknesses in subtests measuring PIQ and relative strengths in VIQ. Figures 2a and 2b depict the cognitive performance on the WAIS-R subtests and summary measures for the WS patients in our cohort in comparison with 1880 normal controls [27]. For the WS cohort, the mean scores were 67.3±8.4 for Full Scale IQ (FSIQ), 71.1±8.2 for Verbal IQ (VIQ), and 65.8±8.3 for Performance IQ (PIQ), when compared to the normal population. In the WS patients, PIQ is significantly less than VIQ (one-tailed paired t-test, n = 65, p = 0.0001). Relative to their overall performance, WS subjects tended to do well in tests of vocabulary (Vocabulary) and abstract reasoning (Similarities, Picture Arrangement), and poorly in tests of numeracy (Arithmetic), visual-spatial (Digit Symbol, Block Design, Object Assembly), and memory (Digit Span); these subtests are described in Table S1. The distinct pattern of our cohort's WAIS-R subtest scores is similar to WS patients in other studies [28].

### Measurement of WS Cognition II: Principal Components Analysis

We next addressed the problem of how to better represent the distinctive pattern of WS WAIS-R subtest scores. We could not use the WAIS-R summary scores, Full Scale IQ (FSIQ), Verbal IQ (VIQ), or Performance IQ (PIQ) for the measurement of WS cognition because they are control-normalized averages of the individual subtest scores and therefore may not be representative of the subtest variability in WS. In the normal population, each subtest is standardized to have a mean of ten and variance of three, clearly different from the extensive variation of the subtests in our WS patients (Fig. 2a). As explained earlier, PCA is capable of capturing the common variability of the subtests with a reduced number of components. The first generated component (PC1) captures the maximum possible common variation. The subsequent components (PC2 through PC11) capture the sequentially next-highest amount of common variation, without overlap. We accordingly extracted the principal components as summary measures of intelligence for correlation against expression levels.

Our WS population of 65 subjects meets minimum requirements for principal components analysis of the correlation matrix (Table S2) [29]. To objectively determine the number of components within our dataset, we used three common methods: (i) a Scree test [30], (ii) a Kaiser's eigenvalue greater than one test [31], and (iii) a parallel analysis using 10,000 column-wise permutations of our dataset [32]. All three methods agree that the first component (PC1) alone adequately summarizes IQ variation in our WS sample accounting for 58% of the total variance (Table S3). Figure 2c and Table S4 convey how each of the various subtests loads on PC1 in WS cases versus on PC1 obtained in normal controls. For both WS cases and normal controls, all subtests are significantly represented in PC1 (lowest loading is 0.65 for WS cases, and 0.64 for normal controls). However, for WS patients PC1 is more strongly influenced by the five subtests: Block Design, Digit Symbol, Picture Arrangement, Picture Completion and Vocabulary. Vocabulary is a subtest in Verbal IQ; the other four are subtests in visual-spatial or Performance IQ. A markedly different pattern is observed in controls, where a larger influence on PC1 is from the five verbal IQ subtests: Vocabulary, Information, Similarities, Comprehension, and Arithmetic. Thus, in general, scores on performance subtests differentiate WS patients more cleanly than do verbal subtests, whereas for controls, scores on verbal subtests differentiate more cleanly. However, use of the appropriate PC1 would be the best differentiator of any linear combination of subtests.

### Correlation of gene expression with PC1

A previous paper [14] used a multiple regression model to reveal that in a larger WS cohort gene levels were not related to age, parent-of-origin of the deletion, or gender. For the current paper, we identified a sub-cohort of 65 WS subjects with gene expression data and WAIS-R subtest scores. We first correlated the summary scores FSIQ, PIQ, and VIQ against gene expression and obtained results which were not significant after Bonferroni correction.

**Figure 3:**
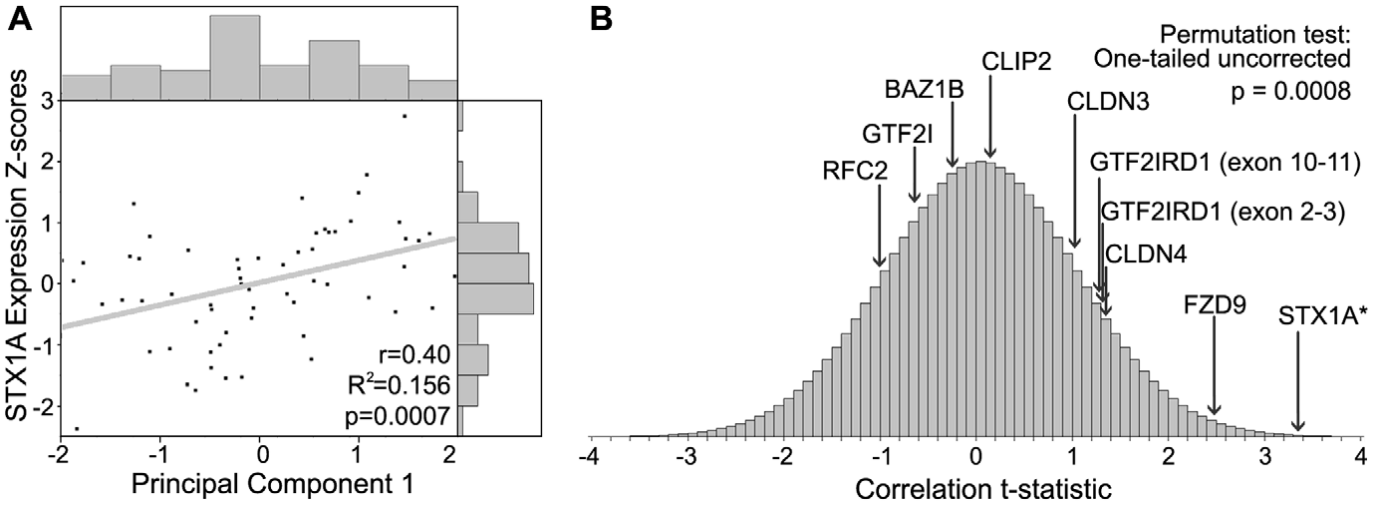
*STX1A* quantitative expression is correlated with intelligence in WS measured by Principal Component 1 of the WAIS-R subtests. **a: Correlation between** ***STX1A*** **expression and PC1.** The Y-axis is Z-scores of *STX1A* expression and the X-axis is PC1 (Pearson correlation p = 0.0007, Bonferroni corrected p = 0.0073). **b: T-ratio distribution of 10^6^ permuted correlations between PC1 and** ***STX1A*** **expression.** In our cohort of 65 WS cases, the t-ratio for PC1 versus the WS genes demonstrates that only the correlation with *STX1A* is not explained by chance alone (Pearson correlation p = 0.0008, Bonferroni corrected p = 0.008).

In order to relate gene expression to cognition, we utilized the data reduction afforded by PCA and tested the correlation of the ten expression levels against PC1 scores (Table 1). In this analysis, PCA is blind to expression levels. Based on the standard one-tailed large sample criterion, we found two significant correlations out of ten tested Pearson correlations (*STX1A* with PC1, one-tailed p = 0.0007; FZD9 with PC1, one-tailed p = 0.0076). Only the correlation between PC1 and *STX1A* remains significant after Bonferroni correction (corrected p = 0.0073) (Table 1). To determine whether PC1 might be related to *STX1A* through other genetic mechanisms in this cohort of 65 WS, we tested the correlation of *STX1A* to age, parent-of-origin of the deletion, and gender. The detailed results are presented in a subsequent section, however we found no significant relationship. Therefore, we conclude that in our data *STX1A* expression is correlated with intelligence as measured by PC1. Since large sample correlation tests are potentially vulnerable to deviations from normality, we also conducted a permutation test of the correlation between *STX1A* and PC1 by permuting PC1 across subjects one million times (SPSS 15.0) (Fig. 3b). Of the one million permutations, only 756 of the computed *STX1A* and PC1 correlations were more extreme in the positive direction than the observed correlation (Bonferroni corrected p = 0.0076). This suggests our results are not due to chance alone. Furthermore, the permutation tests summarized in Figure 3b do not show any other significant correlations between WS genes and PC1 after correction, suggesting a unique role for *STX1A* among the ten tested genes. Finally, we determined that *STX1A* explains 15.6% of PC1 variability in WS patients by regressing PC1 scores out of *STX1A* expression and calculating the percentage reduction in variance (initial variance  = 0.878, final variance  = 0.741). To our knowledge, this is the highest correlation reported between expression of a single gene and intelligence within a human population [2], [3].

**Table 1:** Correlation between quantitative expression of WS genes and intelligence measured by Principal Component 1 (PC1) of the WAIS-R subtests.

		FZD9	BAZ1B	STX1A	CLDN3	CLDN4	RFC2	CLIP2	GTF2IRD1 (2-3)	GTF2IRD1 (10-11)	GTF2I
	**n**	65	65	62	65	65	65	65	65	65	65
	**r**	0.299	-0.030	0.396	0.166	0.126	-0.125	0.021	0.158	0.156	-0.078
	**Asymptotic p-value**	0.008	0.405	0.0007	0.093	0.158	0.162	0.435	0.105	0.107	0.267
	**Corrected asymptotic p-value**	0.077	—	0.0073	0.925	—	—	—	—	—	—
	**Permuted p-value**	0.008	0.405	0.0008	0.092	0.158	0.162	0.436	0.105	0.108	0.268

For each gene, the values listed from top to bottom are: the number of WS subjects with expression levels available (n); the Pearson correlation coefficient (r); the one-tailed asymptotic p-value generated under the assumption of normality (uncorrected for multiple tests); the Bonferroni corrected p-value (or a — if Bonferroni correction results in a value greater than 1.0); and the permutation p-value as shown in Figure 3b (10^6^ permutations; uncorrected for multiple tests). This is shown for *STX1A* in Figure 3a.

### Correlation of gene expression with WAIS-R subtests

The data indicate a correlation between *STX1A* levels and WS intelligence as expressed by PC1, but we sought to determine whether there was a direct correlation with intelligence subtests. To investigate this question, we computed the correlation of each of the ten expression levels against each of the eleven WAIS-R subtests (Table S6). We found 25 significant correlations (one-tailed Pearson correlation, significance threshold p = 0.05) out of 110 tested. Of these, only the correlation between *STX1A* levels and Digit Symbol scores remained significant after Bonferroni correction (corrected p-value = 0.035). Digit Symbol is a multifaceted test measuring multiple skills, including visual memory, spatial memory, motor speed, perceptual processing speed, visual scanning efficiency, and executive function [33]. This makes Digit Symbol a good candidate for a more global measure of intelligence.

### *STX1A* expression is not related to age, parent-of-origin of the deletion, or gender

As mentioned above, to determine whether PC1 might be related to *STX1A* through other established genetic mechanisms, we tested the correlation of *STX1A* to age, parent-of-origin of the chromosome deletion, and gender; we found no significant relationships. PC1 is not related to: subject age (two-tailed Pearson correlation, n = 65, p = 0.4286); subject age at cell line creation (two-tailed Pearson correlation, n = 65, p = 0.3683); subject age at WAIS-R administration (two-tailed Pearson correlation, n = 65, p = 0.3715); age of cell line (two-tailed Pearson correlation, n = 65, p = 0.6735); parent-of-origin of the deletion (two-tailed t-test, n = 55, p = 0.5005); or gender (two-tailed t-test, n = 65, p = 0.8187). *STX1A* expression level is not related to: subject age (two-tailed Pearson correlation, n = 62, p = 0.1063); subject age at cell line creation (two-tailed Pearson correlation, n = 62, p = 0.1032); subject age at WAIS-R (two-tailed Pearson correlation, n = 62, p = 0.1049); age of cell line (two-tailed Pearson correlation, n = 62, p = 0.9404); parent-of-origin of the deletion (two-tailed t-test, n = 53, p = 0.5653); or gender (two-tailed t-test, n = 62, p = 0.9584). Due to a possible weak relationship between subject age and *STX1A* expression, we performed a partial correlation between *STX1A* and PC1 after removing variance added by subject age. The relationship between *STX1A* and PC1 remains highly significant (one-tailed Pearson correlation, n = 62, p = 0.0003).

## Discussion

Nobel Laureate Barbara McClintock once admonished me (JRK), “Let the data speak to you.” Therefore, although unexpected and of unknown mechanism, our data indicate that peripheral *STX1A* expression levels measured in lymphoblastoid cell lines strictly grown, is related to an emergent property of the CNS, intelligence. The cellular and synaptic neurobiology of STX1A is best known as an important component of the presynaptic SNARE complex involved in priming of synaptic vesicles for release. This points to the dosage sensitive modulation of the synapse as one possible aspect of intelligence and to lymphoblastoid cell lines as a possible cellular assay system.

Specifically, our results indicate that *STX1A* is expressed in lymphoblastoid cells, where its transcript level is affected by WS gene deletion and correlated with WS cognition. After Bonferroni correction, the correlation is more significant for PC1 than for PIQ, VIQ, and FSIQ (Table S5), or any single subtest (Table S6). This is not surprising, given that PC1 explains far more of the variation in the WS patients than does any subtest. Taken together, our results are a first step towards dissecting the molecular basis of cognition in WS.

Three other lines of evidence support a role for *STX1A* in learning and memory in WS. First, *STX1A* is largely expressed in the brain regions involved in learning, memory, and fear (cerebral cortex, hippocampus, and amygdala, respectively; http://www.brain-map.org). STX1A is an important component of the synapse, where it localizes at the presynaptic membrane and functions as a part of the Q-SNARE (glutamine soluble N-ethylmaleimide sensitive attachment receptor) complex to regulate neurotransmission through calcium-mediated exocytosis of synaptic vesicles [34], [35]. Moreover, STX1A also interacts with and co-regulates multiple ion channel types and neurotransmitter transporters (e.g., sodium ENaC, potassium KCNB1, and dopamine DAT; Table S7). The second line of evidence is that the *Stx1a* knockout mouse shows impaired hippocampal long-term potentiation and impaired fear memory consolidation and extinction, despite normal brain structure and morphology [36]. Expression of the *STX1A* isoform, *STX1B*, is not elevated in the knockout, suggesting independent function despite co-localization in many but not all brain regions [37]. The third line of evidence is that syntaxin 1A protein levels are increased in autism [38] and decreased in prefrontal cortical neurons in persons with advanced Alzheimer disease (ELISA, t-test p = 0.0003) [39], along with other presynaptic proteins. Combined with our results, these biological clues support a role for *STX1A* in the deficits of learning and memory in WS.

How might *STX1A* expression in lymphoblastoid cell lines be related to neural function? It seems rather extraordinary that our approach indicates *STX1A* expression in LB lines might explain as much as 15.6% of the variance in the first principal component of general intelligence in WS measured by the WAIS-R. As with all new findings, our results need to be replicated in further studies combining cognitive with cellular and genetic analyses. However, one possible explanation is that *STX1A* expression in brain may be the same as that in lymphoid tissue or in lymphoblastoid cells grown under the same conditions, even though it may have no functional consequences in LB lines. Another possibility is that *STX1A* expression in brain may vary with intelligence and perform a cellular role related to that in lymphoblastoid cells. One role STX1A may have in common is SNARE mediating calcium stimulated vesicle fusion both at the neural synapse and at the plasma membrane in non-excitable tissues [40]. Although the role of STX1A in the immune system is less clear, it is involved in exocytosis in some non-excitable tissues, such as insulin secretion by beta cells of the exocrine pancreas and antibody secretion (http://www.genecards.org/index.shtml) [41]. Alternatively, the connection between the peripheral and central role of STX1A may be mediated by immune-nervous system interactions involving both CD4+ and CD8+ lymphocytes that express *STX1A* and enter the central nervous system [42]. Other mechanisms may involve cytokines, known regulators of brain activity, or other similarities of the immune and nervous synapses [43]. It will be of interest to elucidate the possible role of *STX1A* in immune regulation as this may provide insight into cellular pathways in common with the brain [40].

WS is an informative model for dissecting the genetic contributions to human intelligence. Studies of individuals with smaller deletions have already implicated the genes *GTF2IRD1* and *GTF2I* as involved in the characteristic WS deficits in visual-spatial construction [7], [8], [9], [10]. Cognitive deficits remain, however, when these genes are not deleted. It is important to note that a given gene may affect intelligence through multiple causal routes during brain development, post-natal brain function at the synapse, or metabolically. To pinpoint the contributions of other genes to WS cognition we need further genetic sequence or methylation information to identify different subgroups, and to determine if cognition varies among the groups. For example, position effects on genes flanking the deletion may also affect WS. Other genetic mechanisms that may affect WS phenotypes include DNA sequence variations and epigenetic effects originating either from WS genes expressed on the non-deleted chromosome 7 or from the remainder of the genome. Association studies in the normal population designed to detect these effects are limited by the large sample sizes required to overcome the pitfalls of multiple testing. Further limitations of these studies are the large number of genes that undoubtedly influence intelligence and that the effects of any one gene are apt to be small. Finally, if expression levels rather than allele differences are used as predictors of cognitive processes, then inevitable questions for study replication arise concerning the lack of sensitivity and specificity of various array gene expression chips compared to RT-PCR. In contrast, in WS, we have analyzed a large cohort of individuals with sensitive genetic tests to show that variation in a single gene can explain 15.6% of the first principal component of intelligence. Further analyses of DNA sequence and gene expression in this cohort may reveal other genes encoding proteins involved in neuronal synaptic transmission as contributing to intelligence and behavior. Some of these genes may well turn out to be predictive of cognitive function, either in the entire population or perhaps only in those with lower IQ's. Alternatively, *STX1A* may be related to intelligence only in WS. These questions are an important direction for future study.

**Figure 4:**
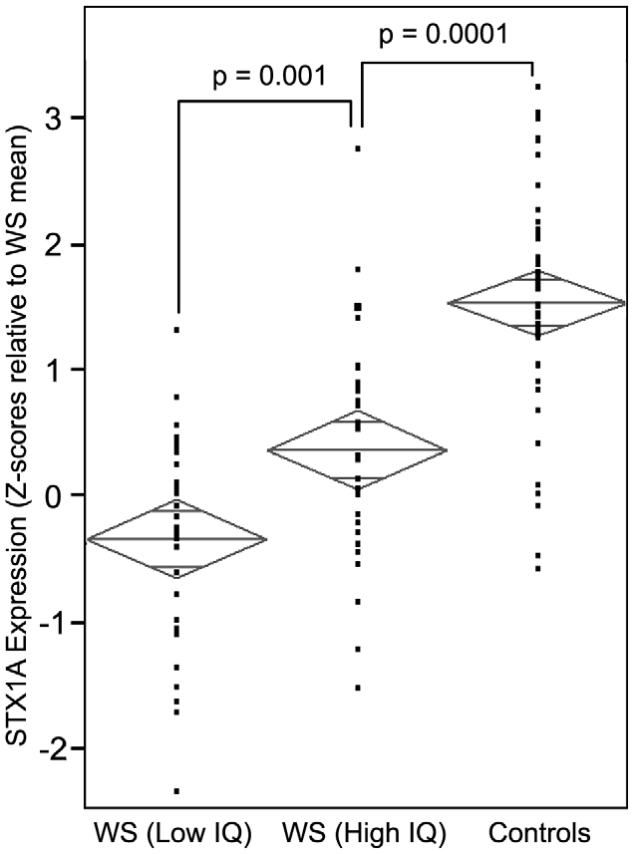
Linear extrapolation between IQ and STX1A levels in WS predicts normal IQ from expression in normal controls. Low IQ WS is defined as cases at or below the median FSIQ for the WS subjects; High IQ WS is at or above the median. The Low IQ WS group (n = 31) had IQ scores 60.13±5.30; the High IQ WS group (n = 31) had IQ scores 74.29±4.09. The middle line across each diamond is the group mean Z-score for *STX1A* expression. The vertical span of each diamond represents the 95% confidence interval for each group. The mean ± standard deviation of *STX1A* expression for Low IQ WS is −0.36±0.85; for High IQ WS it is 0.35±0.90; and for normal controls it is 1.52±0.90. P-values are derived from a two-tailed Student's t-test. Simple linear extrapolation then implies that the *STX1A* expression levels in the normal controls (n = 45) predict a mean IQ from 91.1 (*ACTB* normalized expression) to 105.0 (*PPIA* normalized expression). These data suggest that *STX1A* expression may be related to variability in IQ in WS and in the normal population. This conclusion merits further study but could provide a peripheral assay system that reflects vesicle fusion functioning at the synapse. It will be of interest to examine, with sensitive QRT-PCR methods, other genes encoding synaptic proteins, as these become similar candidates for neurocellular processes.

The most profitable place to start preliminary investigation of intelligence in the normal population may be with *STX1A* itself. For example, simple linear extrapolation of the relationship between IQ and *STX1A* in WS predicts normal IQ from normal *STX1A* expression (Fig. 4). In particular, the *STX1A* expression in our 45 normal controls predicts a mean IQ of 91.1 (*ACTB* normalized expression) to 105.0 (*PPIA* normalized expression). In future studies, it will be important to further test this correlation in the normal population, using either *STX1A* gene variation or expression.

To our knowledge, this is one of the first links between a cellular process and an emergent property of neural circuits. Further progress in dissecting the genetics and neurobiology of human cognition will require similar integrative research. The study of WS opens a unique window into this complex world. With good luck and hard work, similar analyses of neurodevelopmental disorders may reveal more of Nature's hand in shaping intelligence at the synapse.

## Materials and Methods

### Patient Population, Ethics Statement, and Data Analysis

Our population of 65 WS patients includes 34 females and 31 males with average age at cell line creation of 28.7±9.0 years. This study was conducted in accordance with the principles expressed in the Declaration of Helsinki. The subjects and their families were recruited as part of an ongoing research study approved by the Institutional Review Boards at Cedars-Sinai Medical Center (CSMC) or The Salk Institute. All patients provided written informed consent for the collection of samples and subsequent analysis. In each case the diagnosis of WS was determined by medical history, clinical studies, and laboratory analysis using FISH (fluorescence in situ hybridization). Multicolor FISH then confirmed diagnosis using a panel of well-characterized BACs from both the deleted and flanking duplicated regions as previously described in detail [8]. In typical WS, where single copy BACs for the elastin locus (592D8) or those flanking *STX1A* are deleted (including 1008H17 and 315H11 centromeric, and 1184P14 telomeric), the region containing *STX1A* is included in the deletion, as are regions identified by BACs for the duplicated regions (including 239C10). In order to validate the deletion of *STX1A* in WS subjects, quantitative PCR using TaqMan assay primer sets (Applied Biosystems, USA) on the DNA extracted from the lymphoblastoid cells lines were performed on 65 WS subjects and 10 normal parental controls. Standardized TaqMan RNase P Control (VIC) reagents were used as the endogenous reference (2 copies) in multiplex reactions. A custom TaqMan assay for *STX1A* was designed with the forward primer as 5′-ACCCTCAAAACGGTTCATTCGT-3′, the reverse primer as 5′-CCAGGTTCAGTGCTCTTTCACA-3′, and the FAM reporter sequence as 5′-CTGGCTCAGCAGCTCC-3′. All WS subjects completed the WAIS-R test [5] and donated cells for quantitative gene expression analysis. The WAIS-R testing was conducted largely at the same time as the blood draw for LB cell line generation (on the same day for 42 subjects, within 2 years for an additional 18 subjects, and within 4–8 years for the remaining 5 subjects, all of whom were 21–46 years old). All cell lines were generated at CSMC using standard methods.

All correlations performed are Pearson's correlations. We used SPSS 15.0 (www.spss.com) to perform principal components analysis and permutation analysis. All other statistical analyses were performed using JMP 7 (www.jmp.com).

## Supporting Information

Figure S1 Gene expression level measured by quantitative RT-PCR [14] in WS subjects and normal controls. Expression levels measured by quantitative RT-PCR of lymphoblast cDNA of genes FZD9 (n = 65 WS subjects), BAZ1B (65), STX1A (62), CLDN3 (65), CLDN4 (65), RFC2 (65), CLIP2 (65), GTF2IRD1 (exon 2-3) (65), GTF2IRD1 (exon 10-11) (65), and GTF2I (65) in WS subjects (dark grey) and normal controls (light grey) are graphed as z-scores relative to the WS mean.
(16.88 MB TIF)

Figure S2 The deletion of STX1A was confirmed by quantitative PCR in 65 WS subjects and 10 normal controls. A custom TaqMan assay for STX1A was used with standardized TaqMan RNase P Control (VIC) reagents as the endogenous reference. The mean relative copy number of STX1A is 1.05±0.15 in WS (n = 65, solid diamonds) and 1.96±0.14 in normal controls (n = 10, solid dots).
(3.99 MB TIF)

Table S1 WAIS-R subtest descriptions [5, S2].
(0.04 MB DOC)

Table S2 WAIS-R subtest correlation matrix (R2 values) in WS cases (n = 65) and in normal controls. Lower triangle (italics) represents correlations in WS cases; upper triangle represents correlations in normal controls [5]. Performance subtests are listed in bold; verbal subtests in plain font.
(0.05 MB DOC)

Table S3 Proportion of variance in WAIS-R subtests explained by PCA Components 1-11 in our WS cohort. Component 1 alone explains 57.6% of the variance in WAIS-R subtests.
(0.04 MB DOC)

Table S4 WAIS-R subtest loadings on the first principal component in WS cases and in normal controls. Performance subtests are listed in bold; verbal subtests in plain font. Loadings for normal controls are derived from Enns and Reddon [27].
(0.03 MB DOC)

Table S5 Correlation between quantitative expression of WS genes and WAIS-R VIQ, PIQ, and FSIQ in WS cases. For each gene and test, the top number is the Pearson correlation coefficient (r) and the bottom number is the one-tailed p-value (uncorrected for multiple tests).
(0.05 MB DOC)

Table S6 Correlation between quantitative expression of WS genes and WAIS-R subtest scores in WS cases. For each gene and test, the top number is the Pearson correlation coefficient (r) and the bottom number is the one-tailed p-value (uncorrected for multiple tests).
(0.09 MB DOC)

Table S7 Syntaxin 1A binds to and regulates multiple ion channels and neurotransmitter transporters. STX1A performs this function in addition to its role in presynaptic vesicle processing.
(0.04 MB DOC)
