# Supplementary figures and images for: Intelligence in Williams Syndrome Is Related to STX1A, Which Encodes a Component of the Presynaptic SNARE Complex
Source: PLoS One. 2010 Apr 21;5(4):e10292. doi: 10.1371/journal.pone.0010292 (PMC2858212; doi:10.1371/journal.pone.0010292)

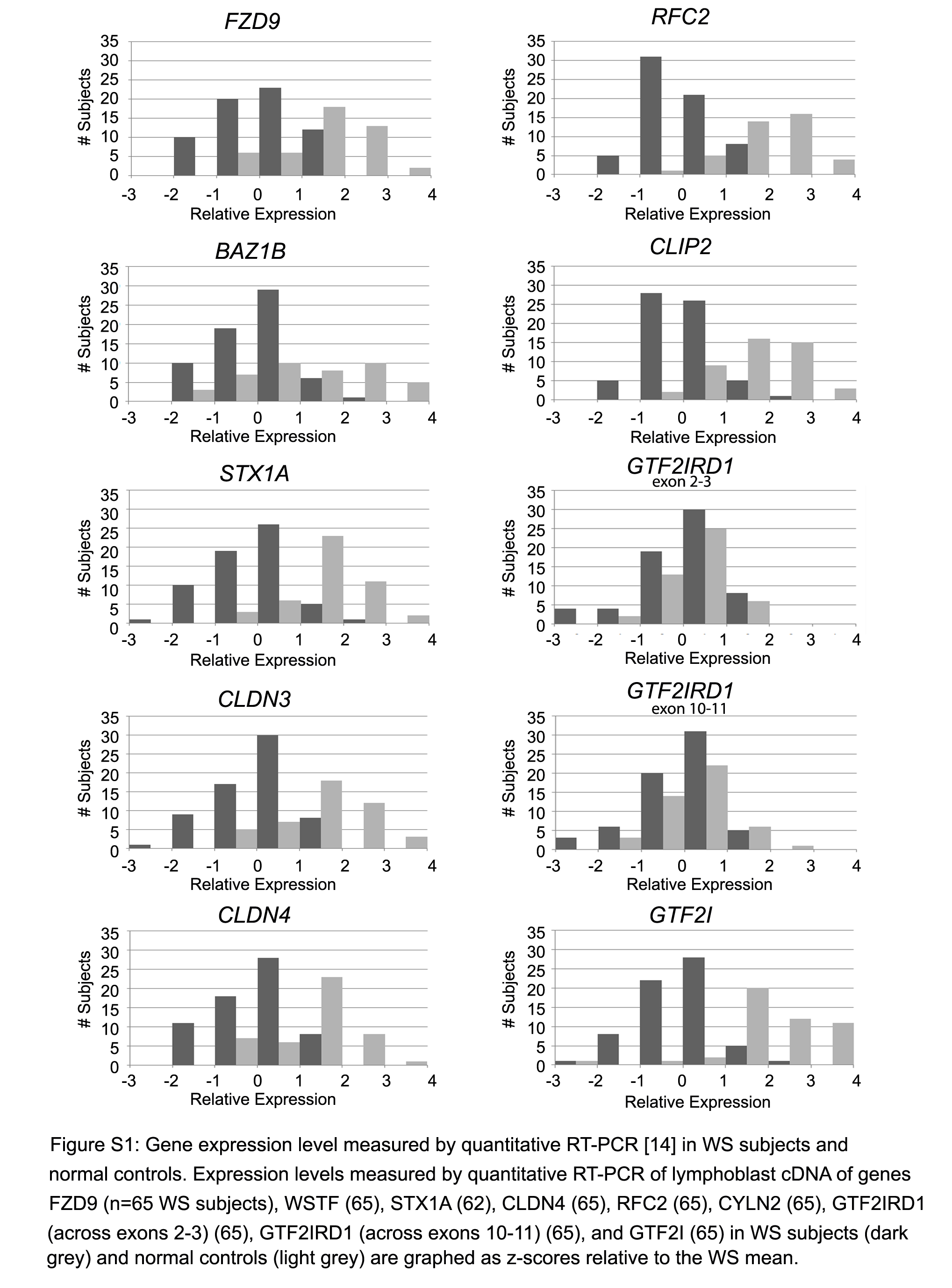

Supplement: Figure S1 — Gene expression level measured by quantitative RT-PCR [14] in WS subjects and normal controls. Expression levels measured by quantitative RT-PCR of lymphoblast cDNA of genes FZD9 (n = 65 WS subjects), BAZ1B (65), STX1A (62), CLDN3 (65), CLDN4 (65), RFC2 (65), CLIP2 (65), GTF2IRD1 (exon 2-3) (65), GTF2IRD1 (exon 10-11) (65), and GTF2I (65) in WS subjects (dark grey) and normal controls (light grey) are graphed as z-scores relative to the WS mean. (16.88 MB TIF) [file pone.0010292.s001.tif]

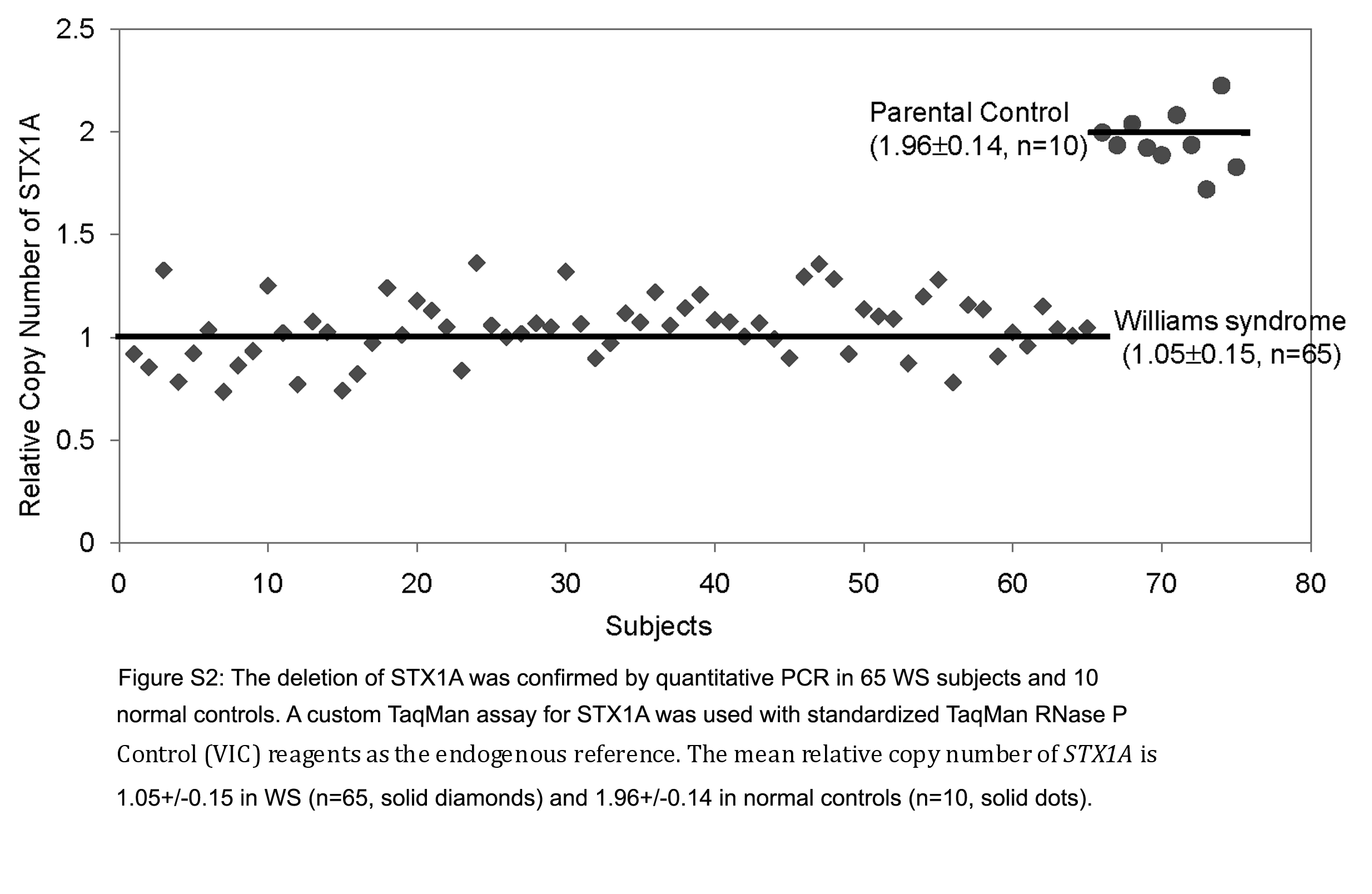

Supplement: Figure S2 — The deletion of STX1A was confirmed by quantitative PCR in 65 WS subjects and 10 normal controls. A custom TaqMan assay for STX1A was used with standardized TaqMan RNase P Control (VIC) reagents as the endogenous reference. The mean relative copy number of STX1A is 1.05±0.15 in WS (n = 65, solid diamonds) and 1.96±0.14 in normal controls (n = 10, solid dots). (3.99 MB TIF) [file pone.0010292.s002.tif]
