# Supplementary material for: Intelligence in Williams Syndrome Is Related to STX1A, Which Encodes a Component of the Presynaptic SNARE Complex
Source: PLoS One. 2010 Apr 21;5(4):e10292. doi: 10.1371/journal.pone.0010292 (PMC2858212; doi:10.1371/journal.pone.0010292)
Supplement: Table S1 — WAIS-R subtest descriptions [5, S2]. (0.04 MB DOC) [file pone.0010292.s003.doc]

**Table S1: WAIS-R subtest descriptions [5, S2].**

| **Summary Tests** | **Description** |
| --- | --- |
| Full Scale IQ | Age-scaled sum of all subtest scores. |
| Verbal IQ | Age-scaled sum of verbal subtest scores. |
| Performance IQ | Age-scaled sum of all performance subtest scores. |
|  |  |
| **Verbal Subtests** | **Description** |
| Vocabulary | List of orally and visually printed words; the examinee is required to provide oral definitions. |
| Similarities | Orally presented pairs of words; examinee explains the similarity between two words or concepts. |
| Information | Orally presented questions related to common events, objects, places, and people. |
| Comprehension | Series of orally presented questions related to social rules and concepts or solutions to everyday problems. |
| Arithmetic | Series of orally presented arithmetic problems; examinee must solve them mentally and express them orally. |
| Digit Span | Series of orally presented letters and numbers; examinee mentally tracks these and orally presents them with the numbers in ascending order and the letters in alphabetical order. |
|  |  |
| **Performance Subtests** | **Description** |
| Picture Arrangement | Cartoon-type pictures presented in a mixed-up order; examinee must rearrange them to make a logical story sequence. |
| Picture Completion | Set of color pictures or common objects and settings, each picture is missing an important part; the examinee is requested to identify the most important part that is missing. |
| Block Design | Set of blocks; examinee must arrange the blocks to replicate various patterns. |
| Object Assembly | Set of puzzles of common objects; examinee assembles the pieces. |
| Digit Symbol | Examinee must match and write down the symbol which corresponds with the number. |
