# Supplementary material for: Intelligence in Williams Syndrome Is Related to STX1A, Which Encodes a Component of the Presynaptic SNARE Complex
Source: PLoS One. 2010 Apr 21;5(4):e10292. doi: 10.1371/journal.pone.0010292 (PMC2858212; doi:10.1371/journal.pone.0010292)
Supplement: Table S2 — WAIS-R subtest correlation matrix (R2 values) in WS cases (n = 65) and in normal controls. Lower triangle (italics) represents correlations in WS cases; upper triangle represents correlations in normal controls [5]. Performance subtests are listed in bold; verbal subtests in plain font. (0.05 MB DOC) [file pone.0010292.s004.doc]

**Table S2**: **WAIS-R subtest correlation matrix (R^2^ values) in WS cases (n=65) and in normal controls.** Lower triangle (italics) represents correlations in WS cases; upper triangle represents correlations in normal controls [5]. Performance subtests are listed in bold; verbal subtests in plain font.

|  | **Picture Completion** | **Picture Arrangement** | **Object Assembly** | **Digit Symbol** | **Block Design** | Vocabulary | Similarities | Information | Digit Span | Comprehension | Arithmetic |
| --- | --- | --- | --- | --- | --- | --- | --- | --- | --- | --- | --- |
| Arithmetic | 0.48 | 0.46 | 0.42 | 0.45 | 0.56 | 0.63 | 0.56 | 0.61 | 0.56 | 0.57 | — |
| Comprehension | 0.52 | 0.48 | 0.40 | 0.44 | 0.40 | 0.74 | 0.68 | 0.68 | 0.68 | — | *0.49* |
| Digit Span | 0.37 | 0.37 | 0.33 | 0.63 | 0.56 | 0.46 | 0.45 | 0.46 | — | *0.51* | *0.60* |
| Information | 0.52 | 0.50 | 0.39 | 0.50 | 0.54 | 0.81 | 0.66 | — | *0.60* | *0.41* | *0.56* |
| Similarities | 0.55 | 0.59 | 0.45 | 0.62 | 0.49 | 0.72 | — | *0.52* | *0.42* | *0.46* | *0.53* |
| Vocabulary | 0.56 | 0.68 | 0.47 | 0.56 | 0.55 | — | *0.52* | *0.65* | *0.57* | *0.61* | *0.60* |
| **Block Design** | 0.54 | 0.47 | 0.63 | 0.47 | — | *0.53* | *0.48* | *0.53* | *0.54* | *0.39* | *0.51* |
| **Digit Symbol** | 0.42 | 0.39 | 0.38 | — | *0.61* | *0.54* | *0.61* | *0.49* | *0.62* | *0.60* | *0.55* |
| **Object Assembly** | 0.52 | 0.39 | — | *0.50* | *0.72* | *0.47* | *0.45* | *0.35* | *0.31* | *0.35* | *0.35* |
| **Picture Arrangement** | 0.51 | — | *0.44* | *0.58* | *0.66* | *0.67* | *0.58* | *0.59* | *0.53* | *0.43* | *0.51* |
| **Picture Completion** | — | *0.69* | *0.55* | *0.59* | *0.63* | *0.55* | *0.55* | *0.67* | *0.47* | *0.45* | *0.51* |
