# Supplementary material for: Intelligence in Williams Syndrome Is Related to STX1A, Which Encodes a Component of the Presynaptic SNARE Complex
Source: PLoS One. 2010 Apr 21;5(4):e10292. doi: 10.1371/journal.pone.0010292 (PMC2858212; doi:10.1371/journal.pone.0010292)
Supplement: Table S3 — Proportion of variance in WAIS-R subtests explained by PCA Components 1-11 in our WS cohort. Component 1 alone explains 57.6% of the variance in WAIS-R subtests. (0.04 MB DOC) [file pone.0010292.s005.doc]

**Table S3**: **Proportion of variance in WAIS-R subtests explained by PCA Components 1-11 in our WS cohort.** Component 1 alone explains 57.6% of the variance in WAIS-R subtests.

| **Principal Component** | **Eigenvalue** | **Variance Explained (%)** | **Cumulative Variance Explained (%)** |
| --- | --- | --- | --- |
| 1 | 6.334 | 57.582 | 57.582 |
| 2 | 0.963 | 8.755 | 66.337 |
| 3 | 0.725 | 6.594 | 72.931 |
| 4 | 0.602 | 5.472 | 78.404 |
| 5 | 0.536 | 4.871 | 83.274 |
| 6 | 0.477 | 4.337 | 87.612 |
| 7 | 0.412 | 3.744 | 91.356 |
| 8 | 0.352 | 3.201 | 94.557 |
| 9 | 0.250 | 2.269 | 96.826 |
| 10 | 0.219 | 1.995 | 98.821 |
| 11 | 0.130 | 1.179 | 100.000 |
