# Supplementary material for: Intelligence in Williams Syndrome Is Related to STX1A, Which Encodes a Component of the Presynaptic SNARE Complex
Source: PLoS One. 2010 Apr 21;5(4):e10292. doi: 10.1371/journal.pone.0010292 (PMC2858212; doi:10.1371/journal.pone.0010292)
Supplement: Table S4 — WAIS-R subtest loadings on the first principal component in WS cases and in normal controls. Performance subtests are listed in bold; verbal subtests in plain font. Loadings for normal controls are derived from Enns and Reddon [27]. (0.03 MB DOC) [file pone.0010292.s006.doc]

**Table S4: WAIS-R subtest loadings on the first principal component in WS cases and in normal controls.** Performance subtests are listed in bold; verbal subtests in plain font. Loadings for normal controls are derived from Enns and Reddon [27].

|  | **WS Loadings** | **NC Loadings** |
| --- | --- | --- |
| Arithmetic | 0.74 | 0.78 |
| Comprehension | 0.68 | 0.80 |
| Digit Span | 0.74 | 0.65 |
| Information | 0.77 | 0.82 |
| Similarities | 0.73 | 0.81 |
| Vocabulary | 0.81 | 0.86 |
| **Block Design** | 0.79 | 0.74 |
| **Digit Symbol** | 0.80 | 0.64 |
| **Object Assembly** | 0.65 | 0.64 |
| **Picture Arrangement** | 0.81 | 0.68 |
| **Picture Completion** | 0.80 | 0.73 |
