# Supplementary material for: Intelligence in Williams Syndrome Is Related to STX1A, Which Encodes a Component of the Presynaptic SNARE Complex
Source: PLoS One. 2010 Apr 21;5(4):e10292. doi: 10.1371/journal.pone.0010292 (PMC2858212; doi:10.1371/journal.pone.0010292)
Supplement: Table S5 — Correlation between quantitative expression of WS genes and WAIS-R VIQ, PIQ, and FSIQ in WS cases. For each gene and test, the top number is the Pearson correlation coefficient (r) and the bottom number is the one-tailed p-value (uncorrected for multiple tests). (0.05 MB DOC) [file pone.0010292.s007.doc]

**Table S5: Correlation between quantitative expression of WS genes and WAIS-R VIQ, PIQ, and FSIQ in WS cases.** For each gene and test, the top number is the Pearson correlation coefficient (r) and the bottom number is the one-tailed p-value (uncorrected for multiple tests).

|  |  | **FZD9** | **BAZ1B** | **STX1A** | **CLDN3** | **CLDN4** | **RFC2** | **CLIP2** | **GTF2IRD1  (2-3)** | **GTF2IRD1  (10-11)** | **GTF2I** |
| --- | --- | --- | --- | --- | --- | --- | --- | --- | --- | --- | --- |
|  | N | 65 | 65 | 62 | 65 | 65 | 65 | 65 | 65 | 65 | 65 |
|  |  |  |  |  |  |  |  |  |  |  |  |
| **Performance IQ** | **r** | **0.218** | **-0.072** | **0.283** | **0.117** | **0.146** | **-0.104** | **-0.122** | **0.126** | **0.105** | **-0.033** |
|  | p | 0.040 | 0.285 | 0.013 | 0.177 | 0.123 | 0.204 | 0.166 | 0.158 | 0.204 | 0.396 |
|  |  |  |  |  |  |  |  |  |  |  |  |
| **Verbal IQ** | **r** | **0.299** | **-0.014** | **0.350** | **0.072** | **0.141** | **-0.172** | **0.081** | **0.197** | **0.154** | **-0.127** |
|  | p | 0.008 | 0.455 | 0.003 | 0.284 | 0.131 | 0.085 | 0.262 | 0.058 | 0.110 | 0.156 |
|  |  |  |  |  |  |  |  |  |  |  |  |
| **Full Scale IQ** | **r** | **0.281** | **-0.055** | **0.336** | **0.086** | **0.130** | **-0.194** | **-0.022** | **0.179** | **0.150** | **-0.106** |
|  | p | 0.012 | 0.330 | 0.004 | 0.249 | 0.150 | 0.061 | 0.432 | 0.077 | 0.117 | 0.201 |
