# Supplementary material for: Intelligence in Williams Syndrome Is Related to STX1A, Which Encodes a Component of the Presynaptic SNARE Complex
Source: PLoS One. 2010 Apr 21;5(4):e10292. doi: 10.1371/journal.pone.0010292 (PMC2858212; doi:10.1371/journal.pone.0010292)
Supplement: Table S6 — Correlation between quantitative expression of WS genes and WAIS-R subtest scores in WS cases. For each gene and test, the top number is the Pearson correlation coefficient (r) and the bottom number is the one-tailed p-value (uncorrected for multiple tests). (0.09 MB DOC) [file pone.0010292.s008.doc]

**Table S6: Correlation between quantitative expression of WS genes and WAIS-R subtest scores in WS cases.** For each gene and test, the top number is the Pearson correlation coefficient (r) and the bottom number is the one-tailed p-value (uncorrected for multiple tests).

|  |  | **FZD9** | **BAZ1B** | **STX1A** | **CLDN3** | **CLDN4** | **RFC2** | **CLIP2** | **GTF2IRD1 (2-3)** | **GTF2IRD1  (10-11)** | **GTF2I** |
| --- | --- | --- | --- | --- | --- | --- | --- | --- | --- | --- | --- |
|  | N | 65 | 65 | 62 | 65 | 65 | 65 | 65 | 65 | 65 | 65 |
|  | | | | | | | | | | | |
| **Arithmetic** | **r** | **0.128** | **0.043** | **0.349** | **0.118** | **0.136** | **-0.099** | **0.132** | **0.137** | **0.033** | **-0.033** |
|  | p | 0.154 | 0.367 | 0.003 | 0.174 | 0.139 | 0.217 | 0.148 | 0.138 | 0.396 | 0.398 |
|  | | | | | | | | | | | |
| **Comprehension** | **r** | **0.370** | **-0.101** | **0.246** | **0.120** | **0.159** | **-0.156** | **0.111** | **0.212** | **0.250** | **-0.077** |
|  | p | 0.001 | 0.211 | 0.027 | 0.170 | 0.103 | 0.107 | 0.189 | 0.045 | 0.022 | 0.270 |
|  | | | | | | | | | | | |
| **Digit Span** | **r** | **0.258** | **0.000** | **0.220** | **0.067** | **0.149** | **-0.125** | **0.043** | **0.096** | **0.113** | **-0.122** |
|  | p | 0.019 | 0.499 | 0.043 | 0.299 | 0.119 | 0.160 | 0.366 | 0.224 | 0.185 | 0.167 |
|  | | | | | | | | | | | |
| **Information** | **r** | **0.231** | **0.088** | **0.288** | **0.017** | **0.063** | **0.029** | **0.145** | **0.194** | **0.143** | **-0.019** |
|  | p | 0.032 | 0.244 | 0.012 | 0.448 | 0.309 | 0.409 | 0.124 | 0.060 | 0.129 | 0.439 |
|  | | | | | | | | | | | |
| **Similarities** | **r** | **0.163** | **-0.040** | **0.316** | **0.053** | **0.092** | **-0.054** | **0.000** | **0.158** | **0.154** | **-0.090** |
|  | p | 0.097 | 0.375 | 0.006 | 0.337 | 0.233 | 0.335 | 0.499 | 0.104 | 0.110 | 0.238 |
|  |  |  |  |  |  |  |  |  |  |  |  |
| **Vocabulary** | **r** | **0.266** | **-0.059** | **0.314** | **0.023** | **0.072** | **-0.353** | **-0.012** | **0.120** | **0.079** | **-0.191** |
|  | p | 0.016 | 0.319 | 0.006 | 0.429 | 0.284 | 0.002 | 0.463 | 0.170 | 0.265 | 0.064 |
|  | | | | | | | | | | | |
| **Block Design** | **r** | **0.263** | **-0.014** | **0.307** | **0.226** | **0.212** | **0.004** | **-0.092** | **0.080** | **0.119** | **-0.002** |
|  | p | 0.017 | 0.455 | 0.008 | 0.035 | 0.045 | 0.489 | 0.234 | 0.263 | 0.172 | 0.494 |
|  | | | | | | | | | | | |
| **Digit Symbol** | **r** | **0.278** | **-0.069** | **0.422** | **0.134** | **0.234** | **-0.160** | **0.137** | **0.153** | **0.178** | **-0.049** |
|  | p | 0.012 | 0.291 | 0.0003 | 0.143 | 0.030 | 0.102 | 0.138 | 0.112 | 0.077 | 0.349 |
|  | | | | | | | | | | | |
| **Object Assembly** | **r** | **0.200** | **-0.079** | **0.378** | **0.092** | **0.208** | **-0.055** | **0.003** | **0.121** | **0.114** | **-0.019** |
|  | p | 0.055 | 0.266 | 0.001 | 0.234 | 0.048 | 0.333 | 0.489 | 0.169 | 0.184 | 0.441 |
|  | | | | | | | | | | | |
| **Picture Arrangement** | **r** | **0.230** | **-0.052** | **0.241** | **0.128** | **0.077** | **-0.079** | **-0.155** | **0.014** | **-0.012** | **-0.080** |
|  | p | 0.033 | 0.342 | 0.030 | 0.155 | 0.270 | 0.266 | 0.109 | 0.456 | 0.461 | 0.264 |
|  | | | | | | | | | | | |
| **Picture Completion** | **r** | **0.124** | **0.019** | **0.252** | **0.075** | **0.010** | **0.013** | **-0.114** | **0.083** | **0.128** | **0.028** |
|  | p | 0.162 | 0.442 | 0.024 | 0.276 | 0.470 | 0.459 | 0.184 | 0.256 | 0.154 | 0.414 |
