# Supplementary material for: Intelligence in Williams Syndrome Is Related to STX1A, Which Encodes a Component of the Presynaptic SNARE Complex
Source: PLoS One. 2010 Apr 21;5(4):e10292. doi: 10.1371/journal.pone.0010292 (PMC2858212; doi:10.1371/journal.pone.0010292)
Supplement: Table S7 — Syntaxin 1A binds to and regulates multiple ion channels and neurotransmitter transporters. STX1A performs this function in addition to its role in presynaptic vesicle processing. (0.04 MB DOC) [file pone.0010292.s009.doc]

**Table S7: Syntaxin 1A binds to and regulates multiple ion channels and neurotransmitter transporters.** STX1A performs this function in addition to its role in presynaptic vesicle processing.

| **Ion** | **Channel** | **Supplemental Reference** |
| --- | --- | --- |
| sodium | ENaC | Condliffe 2004 [S1] |
| chlorine | CFTR | Cormet-Boyaka 2002 [S2] |
| calcium | N-type | Jarvis 2001 [S3] |
| potassium | Kv 1.1, 4.2 | Yamakawa 2007 [S4] |
|  | | |
| **Neurotransmitter** | **Transporter** | **Supplemental Reference** |
| glycine | GLYT2 | Geerlings 2001 [S5] |
| GABA | GAT1 | Wang 2003 [S6] |
| dopamine | DAT | Lee 2004 [S7] |
| norepinephrine | NET | Dipace 2007 [S8] |

**Supplemental References**

1. Condliffe SB, Zhang H, Frizzell RA (2004) Syntaxin 1A regulates ENaC channel activity. J Biol Chem 279: 10085-10092.

2. Cormet-Boyaka E, Di A, Chang SY, Naren AP, Tousson A, Nelson DJ, et al. (2002) CFTR chloride channels are regulated by a SNAP-23/syntaxin 1A complex. Proc Natl Acad Sci U S A 99: 12477-12482.

3. Jarvis SE, Zamponi GW (2001) Distinct molecular determinants govern syntaxin 1A-mediated inactivation and G-protein inhibition of N-type calcium channels. J Neurosci 21: 2939-2948.

4. Yamakawa T, Saith S, Li Y, Gao X, Gaisano HY, Tsushima RG (2007) Interaction of syntaxin 1A with the N-terminus of Kv4.2 modulates channel surface expression and gating. Biochemistry 46: 10942-10949.

5. Geerlings A, Nunez E, Lopez-Corcuera B, Aragon C (2001) Calcium- and syntaxin 1-mediated trafficking of the neuronal glycine transporter GLYT2. J Biol Chem 276: 17584-17590.

6. Wang D, Deken SL, Whitworth TL, Quick MW (2003) Syntaxin 1A inhibits GABA flux, efflux, and exchange mediated by the rat brain GABA transporter GAT1. Mol Pharmacol 64: 905-913.

7. Lee KH, Kim MY, Kim DH, Lee YS (2004) Syntaxin 1A and receptor for activated C kinase interact with the N-terminal region of human dopamine transporter. Neurochem Res 29: 1405-1409.

8. Dipace C, Sung U, Binda F, Blakely RD, Galli A (2007) Amphetamine induces a calcium/calmodulin-dependent protein kinase II-dependent reduction in norepinephrine transporter surface expression linked to changes in syntaxin 1A/transporter complexes. Mol Pharmacol 71: 230-239.
